# Supplementary figures and images for: The inheritance of female colour polymorphism in Ischnura genei (Zygoptera: Coenagrionidae), with observations on melanism under laboratory conditions
Source: PeerJ. 2016 Sep 1;4:e2380. doi: 10.7717/peerj.2380 (PMC5012302; doi:10.7717/peerj.2380)

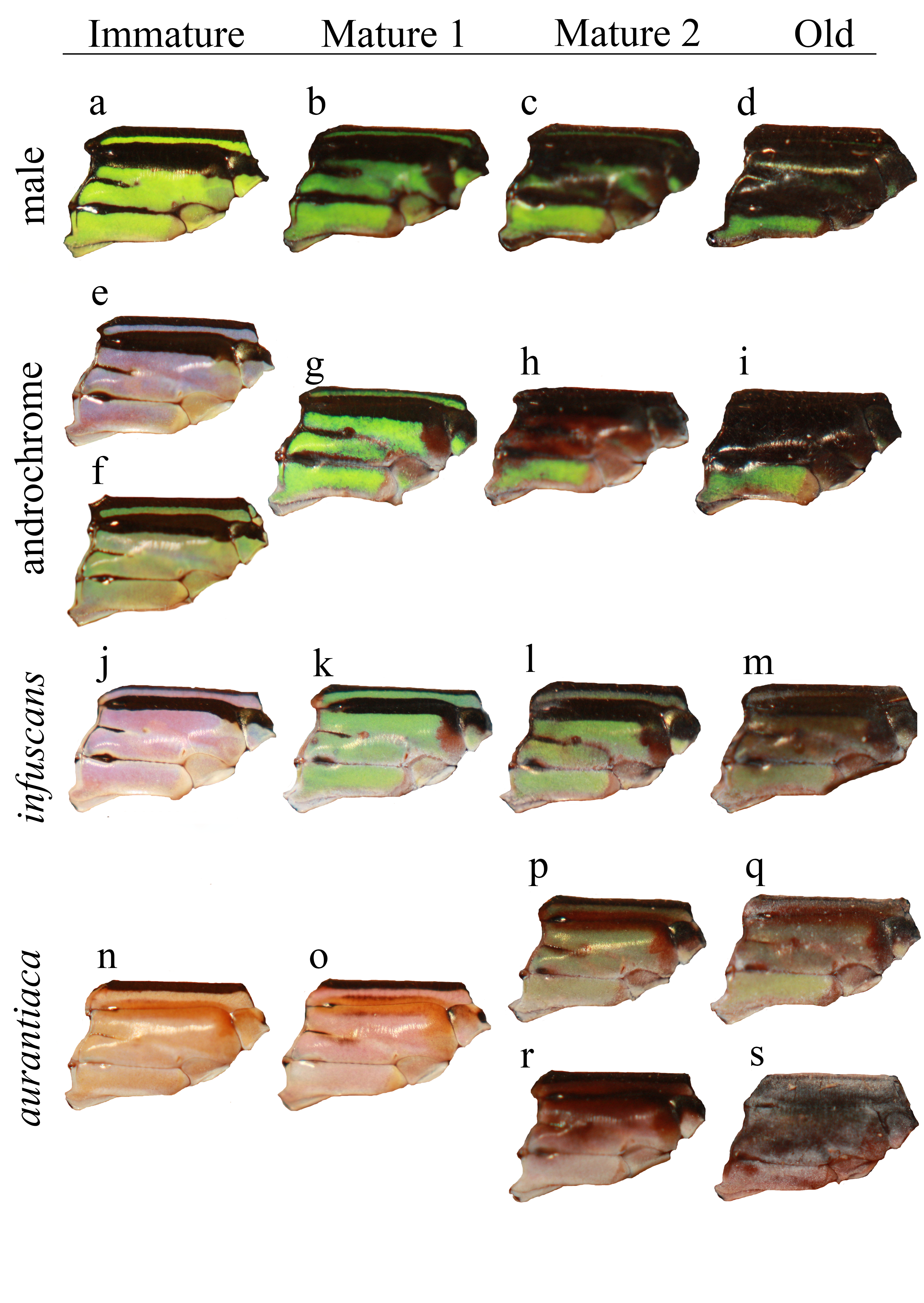

Supplement: Figure S1 — Ontogenic thorax colour change for sex and morph in reared individuals. Thoraxes are positioned in a lateral point of view, with the head to the right and the abdomen on the left. Two thoraxes in the same stage of the same morph (e–f, p–r, q–s) show colour variation and not change. Immature: one day after emergence; Mature 1, early mature individual (5–7 days after emergence); Mature 2, older mature individual (more than 7 days after emergence); Old: individual with more than 15 days. [file peerj-04-2380-s002.png]

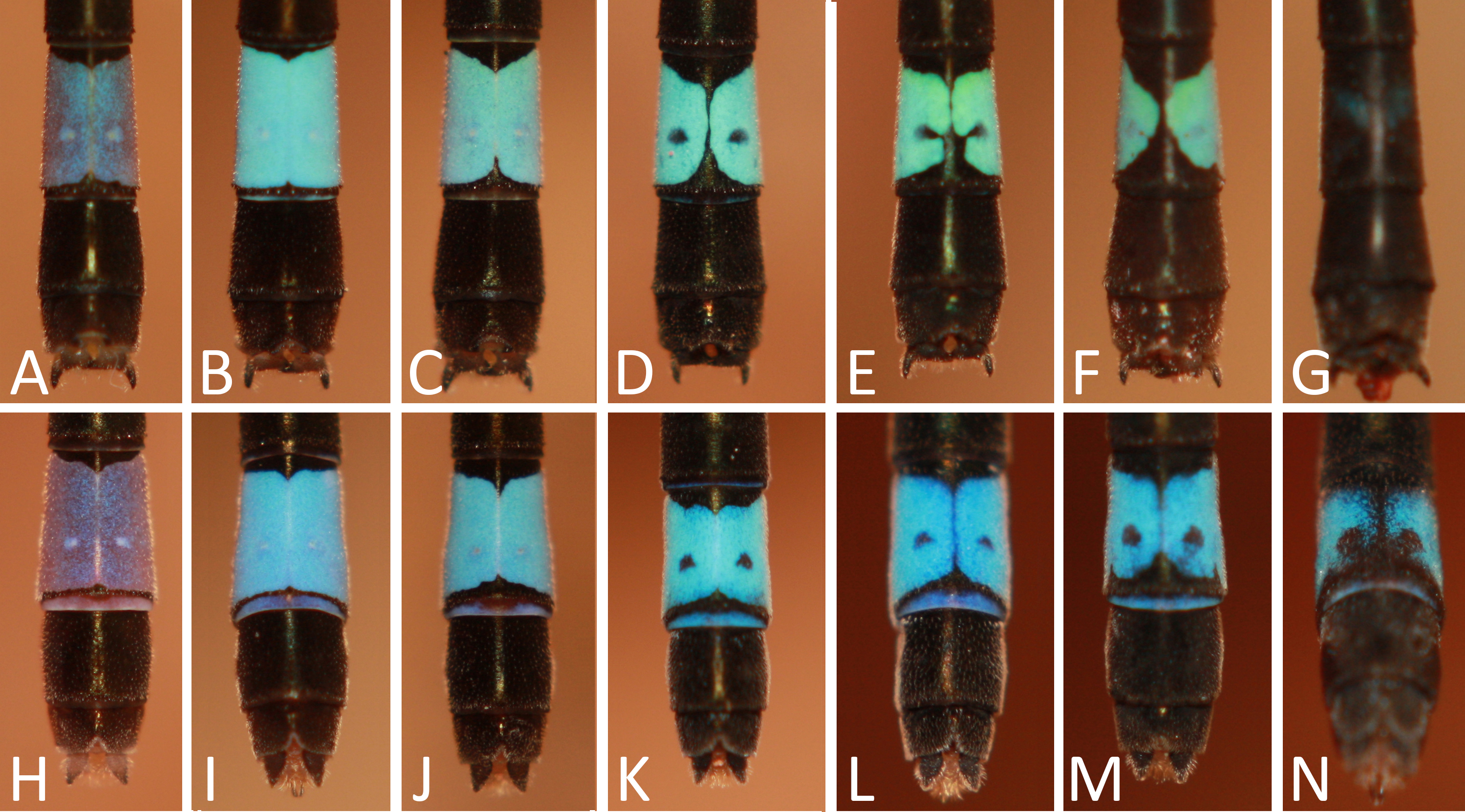

Supplement: Figure S2 — Dorsal view of final abdominal segments showing the pattern variance in reared immature individuals. The last two images of each row correspond to the same individual at different times to appreciate the ontogenic change. Individuals were placed in this order to show the common development of the melanic pattern. Males: upper row. Androchromes: lower row. (A–B): complete blue coloured S8. (C) black colour started to develop as two dorsal arches in the S8 (anterior and posterior) with a peak in the middle towards the inner region of the segment. (D–G) appearance of two symmetrical dots around carinae and three dots in the lateral part of the S8; a longitudinal black stripe connected the superior and inferior black arches due to the elongation of the middle peaks; this stripe became wide in the basal region; the two dots and the basal regions became connected. Not all individuals showed the complete development. Individuals could present the S8 colouration in an advanced pattern one day after emergence and/or maintain one intermediate pattern until death. [file peerj-04-2380-s003.png]
